# Multi-Path U-Net Architecture for Cell and Colony-Forming Unit Image Segmentation

**DOI:** 10.3390/s22030990

**Published:** 2022-01-27

**Authors:** Vilen Jumutc, Dmitrijs Bļizņuks, Alexey Lihachev

**Affiliations:** 1 Institute of Smart Computer Technologies, Riga Technical University, LV-1658 Riga, Latvia; jumutc@gmail.com; 2 Institute of Atomic Physics and Spectroscopy, University of Latvia, LV-1586 Riga, Latvia; aleksejs.lihacovs@lu.lv

**Keywords:** U-Net, skip-connections, neural network, encoder–decoder, Layer Normalization

## Abstract

U-Net is the most cited and widely-used deep learning model for biomedical image segmentation. In this paper, we propose a new enhanced version of a ubiquitous U-Net architecture, which improves upon the original one in terms of generalization capabilities, while addressing several immanent shortcomings, such as constrained resolution and non-resilient receptive fields of the main pathway. Our novel multi-path architecture introduces a notion of an individual receptive field pathway, which is merged with other pathways at the bottom-most layer by concatenation and subsequent application of Layer Normalization and Spatial Dropout, which can improve generalization performance for small datasets. In general, our experiments show that the proposed multi-path architecture outperforms other state-of-the-art approaches that embark on similar ideas of pyramid structures, skip-connections, and encoder–decoder pathways. A significant improvement of the Dice similarity coefficient is attained at our proprietary colony-forming unit dataset, where a score of 0.809 was achieved for the foreground class.

## 1. Introduction

U-Net [1] is arguably the most famous example of an extremely simple (but working) deep learning architecture in the biomedical domain. It uses encoder–decoder pathways alleviated with skip-connections [2] while visually resembling a U-shaped pathway. Many successful applications of the U-Net architecture could be found in cell and nuclei segmentations for digital pathology [3], tumor and organ segmentations [4,5] as well as colony-forming units (CFUs) and other cell segmentation tasks [6,7,8]. This vast diversity of applications has promoted credibility and trustworthiness in the U-Net architecture among researchers.

Despite multiple success stories, segmentation of biomedical images is still far from being a resolved issue. For instance, lesions in medical images demand a higher level of accuracy than what is acceptable in natural images and, thus, it is not possible to rely on a coarse-grained flow of information [9]. Furthermore, CFU segmentation seems to suffer from drifting image acquisition conditions, background noise, an extreme diversity of backgrounds, bacteria types, and possible shapes and textures since agar plates are collected in different labs, environments, and conditions [10]. Microbial cell counting is one of the basic quantitative measurements in microbiology. It relies on the “golden standard”—counting of the CFUs [11]. This counting is an approved standard among procedures used to assess a microbiological contamination of the food and other samples [12]. To detect a colony, a user has to wait until it reaches a certain size to become visible. Typically, 24 h are required to perform the reliable CFU counting; however, by that moment, some of the colonies may have grown and merged too quickly. Therefore, there is a demand for quick automated methods [10,13] to distinguish colonies while they are still small and not merged together. Smaller colonies are harder to detect since they could be easily classified as artifacts, e.g., the food crumbs or working notes on the Petri dish surface.

Existing segmentation algorithms—as described in the following sections—are rendered imprecise and unreliable when a colony or a single cell is overly minuscule. Conventional segmentation algorithms, such as region-based segmentation relying on thresholds or edge-based detection relying on the Sobel or Laplacian operator [14] utilize very simplistic concepts. Among these concepts, we can pinpoint the thresholding on gray-scale information or the presence of discontinuous local features in the image where the most significant part of the image changes in local brightness. Compared to a deep learning U-Net model, these approaches can be considered unsupervised and incapable of capturing nuanced discriminatory features of the foreground class. All factors mentioned above undermine the reliability of the existing segmentation approaches among biologists, practitioners, and food safety experts, since these approaches frequently underperform and only allow segmenting out too few or too many objects. Therefore, it is necessary to develop and test new image segmentation approaches, such as enhanced U-Net architectures, which can efficiently recover the very fine details of the foreground class.

The U-Net was first introduced by Ronneberger et al. [1]. Many improvements have been made since then, which utilize the same backbone architecture, but enhance it with the better building blocks or revisit the structural composition of the main pathway. From the first cohort of works the research by Isensee et al. [15] can be mentioned. The researchers won the KiTS2019 challenge [5] with their enhanced “plain” U-Net architecture. This “plain” approach does not make use of any residual or dense connections [16], it builds upon improving a standard U-Net layer with instance normalization [17], leaky ReLU activations [18], and replicated convolutional layers. On the other hand, Zhou et al. [9] proposed a modified U-Net++ architecture, which utilizes redesigned skip pathways. In the latter approach, feature maps undergo dense blocks of convolutions, whose numbers depend on the pyramid level. In return, this brings the semantic level of the encoder feature maps closer to that of the feature maps awaiting for concatenation in the decoder. While this approach is based on bridging the semantic gap by means of additional skip-connections, Ibtehaz et al. [19] proposed to bolster these connections with the *MultiRes* blocks, resting on the ideas of inception-like architectures. Another interesting proposal by Gao et al. [20] was based on [9] by introducing a covariance self-attention block [21] at the very bottom pyramid level. This block enables a so-called criss-cross attention mechanism [22], which incorporates criss-crossed elements of a feature map (in spatial dimensions) as opposed to flattening feature maps, and then applying an ordinary self-attention calculus. Another promising BRAVE-NET model [23] was recently explored for the brain vessel segmentation problem. The authors apply a multi-scale approach and context aggregation for extending the encoder part of the U-Net with a so-called context path. This path starts with a downsampling by average-pooling with 2×2×2 kernels and a stride of 2, and is concatenated with the main path in the decoder. Finally, unsupervised learning may be considered one more promising approach to solution of segmentation problems. Recently an adaptive squeeze-and-shrink (ASAS) image denoising [24] was proposed for improving a deep detection of cerebral microbleeds. This approach embarks on the ideas of an optimization problem, which comes down to an ASAS operation on the underlying PCA coefficients and can be efficiently coupled with a U-Net architecture to detect cerebral microbleeds.

In order to promote the usage of the U-Net architecture for biomedical image segmentation, a novel multi-path U-Net architecture designed to address many of the aforementioned problems is proposed in this paper. The core proposition is based on two main innovations, i.e., (1) individual receptive field pathways with a consecutive application of Layer Normalization [25] to them, alongside with (2) the introduction of Spatial Dropout [26] to all crucial ramification and concatenation layers. The individual receptive field pathway is the core element of the proposed architecture, it is introduced to allow for the diversification of high- and low-resolution feature maps with a segregated flow of information. The latter is very important if visual information about a segmented-out object is not well-localized in the input space. Furthermore, it is argued that having two or more pathways simplifies model learning and yields faster convergence rates [9]. According to the conducted experiments, multi-path U-Net architecture yields better results than other state-of-the-art approaches, which embark on similar ideas of pyramid structures, skip-connections, and encoder–decoder pathways.

For practical reasons, the proposed approach is compared to the ones by Isensee et al. [15], Zhou et al. [9], Ibtehaz et al. [19], and Kolařík et al. [27] because of inherent similarities and brevity of the format. Any comparisons to the transformer [21], ViT [28,29] architectures, and Gao et al. [20] are intentionally avoided because they comprise a self-attention block, which suggests a completely different and in many cases more complicated outlook on the same segmentation problem.

The remainder of this paper is organized as follows. The proposed methods along with the datasets (materials) and experimental setup are discussed in Section 2, where two core improvements to the U-Net architecture are described in detail. The experimental results are illustrated in Section 3. In Section 4, we discuss the obtained results, pros, and cons of the proposed approach and future work. Finally, Section 5 concludes the paper.

## 2. Materials and Methods

At the beginning of this section, two core innovations to the U-Net architecture are introduced. The datasets used and experimental setup are discussed further, where the performance metrics are presented in order to compare all evaluated approaches.

### 2.1. Multi-Path U-Net

The main proposal set forth in this approach evolves around the idea of individual receptive field pathways with different strides applied for down- and upsampling the spatial dimension. Instead of adjusting the stride of the last convolutional layer as in Isensee et al. [15], the max pooling [30] operation is introduced. All other attributes of the “plain” U-Net architecture are taken intact, e.g., instance normalization [17] and leaky ReLU activations [18]. All receptive field pathways should have coherent strides in order to obtain compatible (in terms of spatial dimensions) feature maps. All feature maps from all pathways are concatenated at the bottom U-Net layer and Layer Normalization [25] is subsequently applied to the output of the aforementioned concatenation. This enables a cross-path normalization operation that effectively intertwines different receptive fields and allows for efficient training and exchange of information.

Formally, the bottom interconnecting layer is defined as follows: let $X_{n}^{i,j}$ denote the *j*-th output feature map from the *i*-th receptive field pathway before the interconnecting layer for the *n*-th example. The final stack $X_{n}^{out}$ of normalized feature maps for the *n*-th example in the training batch can be represented as follows:

(1) $$X_{n}^{out}=\frac{g}{\sigma_{n}}\left({\left[X_{n}^{i,j}\right]}_{i\in [0,k[,j\in [0,l[}-\mu_{n}\right)+b,$$

where *g* and *b* denote, respectively, an adaptive gain and bias learned over all examples jointly, $\mu_{n}$ and $\sigma_{n}$ represent first- and second-order normalization statistics, which are computed across channels/filters (per example) and [] denotes the concatenation layer. All of the above $g,\;b,\;\mu_{n},\;\sigma_{n}$ maintain the spatial dimensions of a single input feature map and are properly broadcasted for the element-wise multiplication and addition. Finally, $\mu_{n}$ and $\sigma_{n}$ are computed as follows:

(2) $$\begin{array}{c} \mu_{n}=\frac{1}{lk}\sum_{i\in [0,k[,j\in [0,l[}X_{n}^{i,j}, \end{array}$$

(3) $$\begin{array}{c} \sigma_{n}=\sqrt{\frac{1}{lk}\sum_{i\in [0,k[,j\in [0,l[}{\left(X_{n}^{i,j}-\mu_{n}\right)}^{2}}. \end{array}$$

### 2.2. Spatial Dropout

In order to enhance the proposed approach, several possibilities have been analyzed to introduce regularization and robustness to the training process. The aforementioned Spatial Dropout [26] has proved to be the best out of many choices. It significantly boosts the generalization performance by promoting independence between feature maps in combination with our novel multi-path U-Net architecture. The decision was made to include the Spatial Dropout as the last layer after all convolution (at the same level of the downsampling path) and deconvolution blocks so that all concatenated feature maps from the upsampling path and skip-connections would have a dropout in place.

### 2.3. Overall Architecture

**Figure 1:**
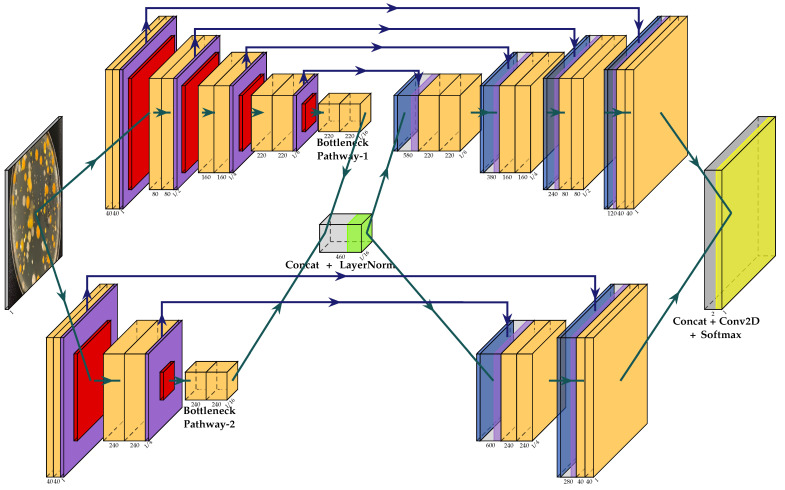
Overall, multi-path U-Net architecture. The input layer is shown in terms of a typical CFU image followed by the ramification into two separate pathways. All convolutional layers are shown in light-orange with the subscripted number of filters, which amounts to a 3×3 kernel, followed by instance normalization and leaky ReLU activation. The Spatial Dropout layer is shown in violet, the max pooling layer in red, and the deconvolutional layer is shown in light-blue. Concatenation layers are depicted in light-gray. Interconnecting and final output layers are captioned and displayed in green and yellow, respectively. All normal pathways are illustrated in green arrow lines, while skip-connections are depicted in blue. Best viewed in color.

The overall multi-path U-Net architecture is presented in Figure 1. For the sake of brevity, the simplest multi-path U-Net model was considered. It has only two receptive field pathways with 2×2 and 4×4 pooling windows and identical strides for the max pooling operation in each respective pathway. All other layers and their corresponding parameters were transferred from the “plain” U-Net architecture, e.g., α=0.1 of the Leaky ReLU function or filter sizes and strides for convolutional layers. The latter is initialized to 3×3 for all filters in all convolution blocks along the downsampling path with the stride being set to s=1. For the upsampling path, deconvolution filter sizes and strides are equal to the receptive field pathway’s max pooling window size. This property ensures that all pathways and skip-connections produce compatible feature maps, which can be concatenated together along the channel/filter axis. The number of filters for the first pathway at each down- and upsampling level was set to [40,240] and [240,40], correspondingly, while the max pooling window was set to 4×4. The second pathway was initialized as follows: [40,80,160,220] for the downsampling path and [220,160,80,40] for the upsampling one, while the max pooling window was set to 2×2. Before proceeding to the interconnecting Layer Normalization, all pathways also have a middle (bottleneck) convolution block, which has either 220 or 240 filters, depending on the pathway. Every convolution block in all pathways consists of two convolutional layers with the number of filters defined above.

It should be noted that different receptive field pathways should have coherent downsampling strides for the Max Pooling operation. More precisely, the number of max pooling operations per pathway is strictly tied to the pooling window sizes. For instance, the aforementioned pathways are coherent as $2^{4}=4^{2}$. Additionally, it should be noticed that the proposed multi-path architecture can easily be extended to three or more paths given that middle (bottleneck) convolution blocks from all the paths are still compatible in size. Due to a hardware resource limitation it was not possible to experiment with higher resolutions and more diverse multi-path architectures comprising of three and more pathways. However, it was assumed that adding more pathways would benefit more difficult image segmentation problems with 4D inputs where receptive fields have to capture an additional dimension.

### 2.4. Datasets

A total of five different cell segmentation datasets were used, out of which four are publicly available. All public datasets are attributed to the Cell Tracking Challenge (2D + Time), which provides datasets for both cell tracking and cell segmentation problems. These datasets comprise the images obtained from mouse hematopoietic and muscle stem cells in hydrogel microwells (BF-C2DL-HSC, BF-C2DL-MuSC), as well as glioblastoma–astrocytoma U373 cells on a polyacrylamide substrate (PhC-C2DL-U373), and HeLa cells stably expressing H2b-GFP (Fluo-N2DL-HeLa). The training counterparts of these datasets were used, where all segmentations were available upfront. The final proprietary dataset comprised colony-forming unit (CFU) images with different types of bacteria being cultured on an agar plate for later segmentation and estimation of the number of cells being initially present. This dataset was collected in-house by our project partner (LTD “Laboratorija Auctoritas”), it comprised 150 images. The latter CFU dataset was annotated by the laboratory staff and trainee students. A brief summary of all datasets is given in Table 1, providing direct links to each dataset (except the proprietary CFU one) with the corresponding characteristics.

### 2.5. Experimental Setup

All architectures were tested under the same experimental setup, envisioning a five-fold cross validation with equally-sized validation sets. During the training stage for each epoch, the model performance on the validation set was tracked and the average of the best attainable Dice similarity coefficient (DSC) [31,32] across all folds was reported. DSC score can be computed as follows:

(4) $$\mathrm{DSC}=\frac{2TP}{2TP+FP+FN},$$

where TP stands for true positives, FP—false positives, and FN—false negatives. All constituents of the aforementioned equation are calculated in a pixel-wise fashion.

**Figure 2:**
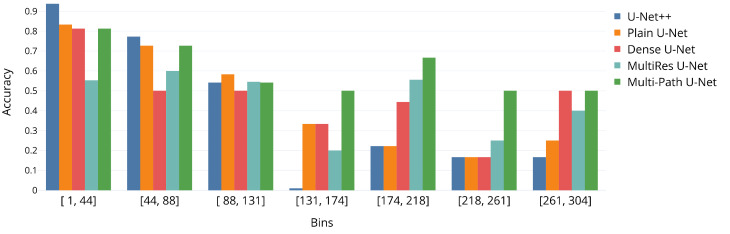
Accuracy of all U-Net models across the first seven bins resulting from the fine-grained binning of CFU counts. Best viewed in color.

For the proprietary CFU dataset, the mean absolute error (MAE) of the CFU counts is reported across all agar plates (images). The other metric of interest for this dataset is the accuracy of binning the CFU counts into three intervals: [0,40), [40,300), and [300,inf). The dynamic programming algorithm was used to keep track of the CFU count; it was applied across all ground-truth and predicted segmentations. Additionally, in Figure 2, the results of our own binning approach, across 20 equally-sized bins, are presented (only the first 7 bins are displayed, as there are enough images to derive the meaningful statistics) in order to gain more insights into the performance with more difficult cases and the working regimes with extremely high CFU counts. This binning approach allows for the direct application of classifiers on the top of the segmentation model for more straightforward usage by biologists and food safety professionals. The choice of three binning intervals is widely accepted in the field of food safety. All cell count verifications were performed under the supervision of our project partner (LTD “Laboratorija Auctoritas”).

The maximum number of epochs was set to 200 and the training was stopped prematurely if the training loss did not improve during 20 consecutive epochs. The sum of Twersky [33] and cross-entropy losses was used as an optimization objective. The former is initialized with α=0.5 and β=0.5, which effectively reduces to a smoothed version of the Dice similarity coefficient. The Adam optimizer [34] was used for training all the models and initial learning rate was set to 0.0001. In the course of training, the learning rate was gradually decreased using time-based decay scheduler. As our initial learning rate was small enough, this did not reduce the final rate too much. All images were re-scaled to 512×512 spatial dimensions using Gaussian smoothing and normalized according to the distribution (pixel-wise z-scores were computed using image mean and standard deviation) before passing them to the segmentation model. For all datasets (except Fluo-N2DL-HeLa), the 3-channel RGB inputs were used, while for Fluo-N2DL-HeLa, only the 16-bit grayscale inputs were available.

Experiments were performed using TensorFlow [35] and MIScnn [36] frameworks. All methods were implemented from scratch using the MIScnn [36] library (except for “plain” U-Net of Isensee et al. [15], which was out-of-the-box available in MIScnn), along with the original Jupyter Notebooks available at GitHub. All code and Jupyter Notebooks were run under a Windows 10 operating system and Anaconda (conda 4.8.3) environment with Python 3.8.8.

## 3. Results

Table 2 presents the experimental results for all datasets as the mean Dice similarity coefficient with standard deviations across all classes (background and foreground) and validation folds. In Table 3, the mean Dice similarity coefficient across all validation folds is provided only for the foreground class. Additionally, the number of model parameters is presented in both Table 2 and Table 3. For the multi-path U-Net architecture, the number of filters in all convolutional and deconvolutional layers was deliberately configured to be on par with “plain” U-Net. The number of filters in U-Net++, Dense U-net, and MultiRes U-Net models was also altered to match closely the aforementioned “plain” U-Net architecture. To provide a proper ablation study outlook in the aforementioned tables, the results of the multi-path U-Net architecture without Spatial Dropout are reported as well. In all other figures and tables “multi-path U-Net” architecture refers only to the complete setup with Spatial Dropout.

The results show that the proposed multi-path approach outperforms the baseline “plain” U-Net, U-Net++, Dense U-Net, and MultiRes U-Net architectures for all datasets. This can be attributed to a novel prospect of individual receptive field pathways and an enhanced wiring thereof, which provides a better flow of visual information (through a different order of resolutions in each pathway) especially when segmented-out object is not well-localized in the input space. Compared to U-Net++ and MultiRes U-Net architectures, the proposed approach significantly boosts the performance and implies that having only redesigned skip-connections with one backbone pathway might not be sufficient for tackling some challenging cell segmentation problems. It should be also noted that U-Net++ and MultiRes models significantly underperform on BF-C2DL-HSC and BF-C2DL-MuSC datasets where segmented out regions of interest are scarce and extremely small compared to the overall image size.

It is important to note that the proposed multi-path model is also considerably more accurate with respect to CFU images with very large number of segmented out colonies. This is clearly observable in Figure 2, where the suggested method outperforms other approaches by a large margin in terms of overall accuracy per bin for the number of colonies over 130.

**Figure 3:**
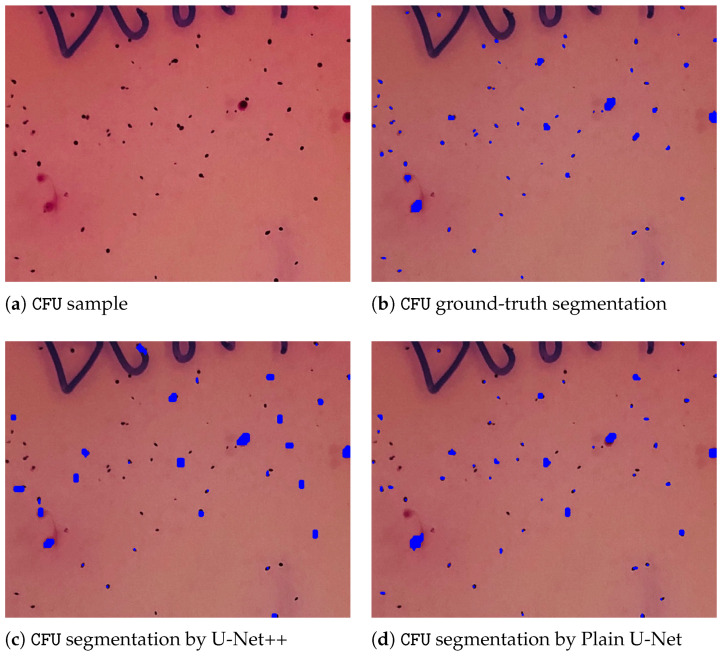

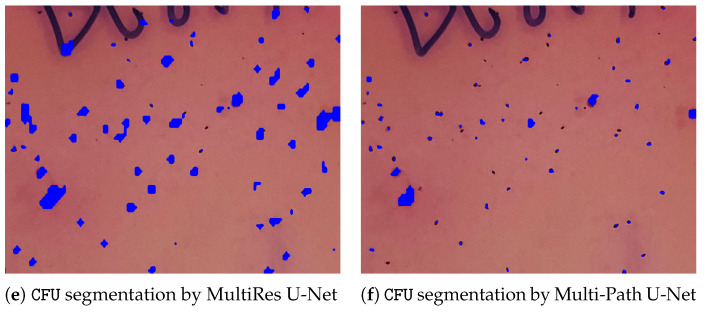
CFU zoomed-in region on a Petri dish with small colonies, ground-truth, and different segmentations obtained from all considered U-Net architectures. (**a**) Contains an unaltered image. (**b**) Represents a ground-truth segmentation annotated manually by our partners. (**c**) Denotes segmentations predicted by the U-Net++ model. (**d**) Denotes segmentations by the plain U-Net model. (**e**) Denotes segmentations by the MultiRes U-Net model. Finally CFU segmentations predicted by the multi-path U-Net model are given in (**f**). As it may be noticed, multi-path U-Net architecture captures, in a fine-grained way, either non-detected (by U-Net++, Plain U-Net) or over-detected (by MultiRes U-Net) CFUs. Best viewed in color.

**Figure 4:**
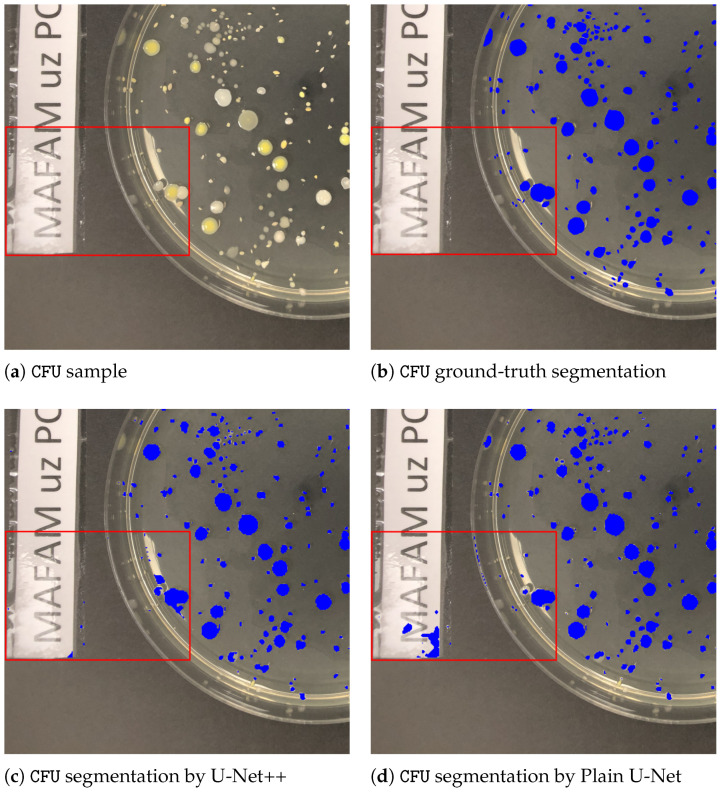

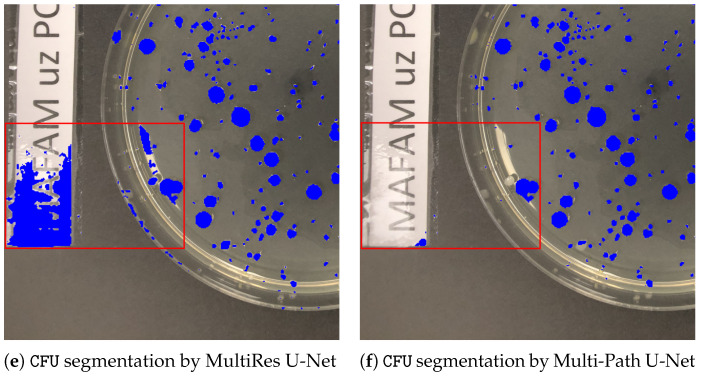
CFU sample on a Petri dish with ground-truth and different segmentations obtained from all considered U-Net architectures. The red bounded box highlights the region with the biggest number of misclassified artifacts (e.g., reflections and captions). (**a**) Contains an unaltered Petri dish image. (**b**) Represents a ground-truth segmentation annotated manually by our partners. (**c**) Denotes segmentations predicted by the U-Net++ model. (**d**) Denotes segmentations by the plain U-Net model. (**e**) Denotes segmentations by the MultiRes U-Net model. Finally CFU segmentations predicted by the multi-path U-Net model are given in (**f**). It may be noticed that multi-path U-Net architecture much better escapes challenging artifacts. Best viewed in color.

**Figure 5:**
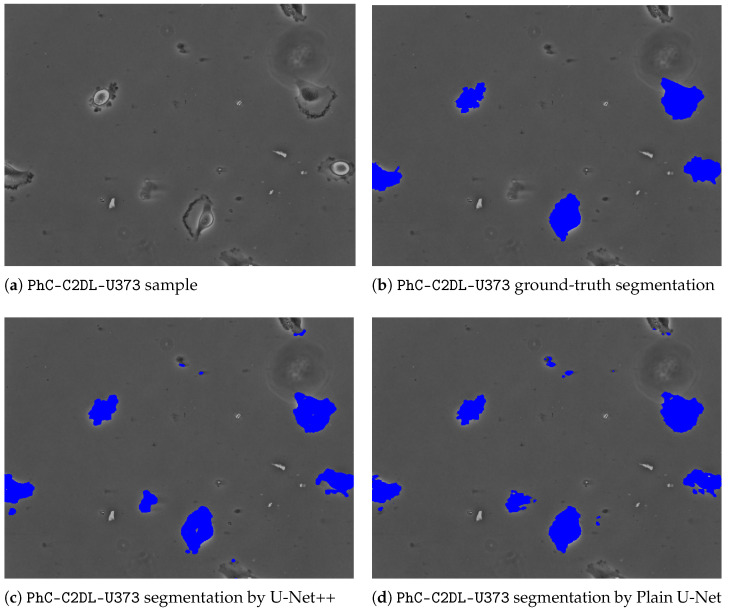

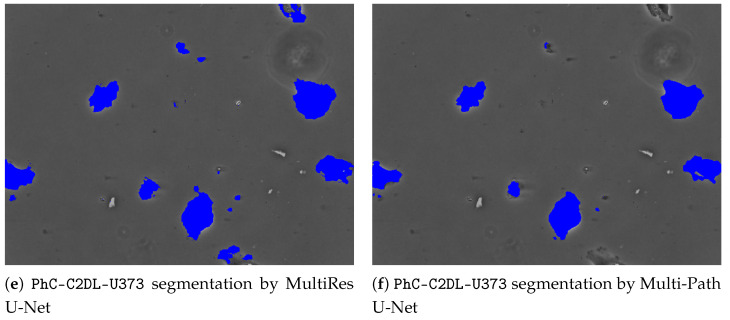
PhC-C2DL-U373 sample with ground-truth and different segmentations obtained from all considered U-Net architectures. (**a**) Contains an unaltered image with differently shaped glioblastoma-astrocytoma U373 cells. (**b**) Represents a ground-truth segmentation available from the Cell Tracking Challenge. (**c**) Denotes segmentations predicted by the U-Net++ model. (**d**) Denotes segmentations by the plain U-Net model. (**e**) Denotes segmentations by the MultiRes U-Net model. Finally, cell segmentations predicted by the multi-path U-Net model are given in (**f**). Best viewed in color.

To give further insights and underpin the findings made, a comparative outlook on the CFU segmentation results from all evaluated models on the images with small CFUs is presented in Figure 3. The latter Figure 3 represents a zoomed-in region with small colonies, ground-truth, and different segmentations obtained from all considered U-Net architectures. As it can be noticed, the proposed multi-path U-Net architecture captures CFUs that are either non-detected (by U-Net++, “plain” U-Net) or over-detected (by MultiRes U-Net) in a fine-grained way. Figure 4 presents different model segmentations with high sensitivity to artifacts (e.g., reflections and captions). This figure represents a Petri dish sample with differently sized CFUs along with text and lighting artifacts. As it may be observed, the multi-path U-Net architecture outperforms other approaches on these challenging artifacts as well. Figure 5 presents segmentation results on a very different dataset, namely PhC-C2DL-U373 (glioblastoma-astrocytoma U373 cells on a polyacrylamide substrate). The latter figure contains differently shaped U373 cells as well as some substrate artifacts. Figure 5 demonstrates that the multi-path U-Net model delivers more accurate segmentations and outperforms other approaches. All segmentations presented in Figure 3, Figure 4 and Figure 5 were obtained from the evaluations on the validation dataset.

To further substantiate the claims regarding the superiority of the multi-path U-Net architecture, additional, MAE and accuracy scores are presented in Table 4. The best MAE score is attained by the considered multi-path architecture. It should also be highlighted that the accuracy score is of immense importance in the food safety applications. As it can be seen, considering the more fine-grained binning of CFU counts, the proposed method indeed outperforms other approaches by at least 6%. Hence, it can be implied that the proposed multi-path U-Net model is highly suitable for CFU classification problems where an improved U-Net architecture might ensure much more accurate early-stage detection of the expired and contagious food samples.

## 4. Discussion

This paper discussed a novel approach to a ubiquitous U-Net architecture for solution of biomedical cell segmentation problems. The proposed multi-path U-Net model embarks on the idea of individual receptive field pathways with different resolutions provided by differently-strided max pooling operations. At the very bottom layer, all the pathways are intertwined using Layer Normalization. To strengthen the proposed architecture and provide higher generalization capabilities, it has been proposed to include the Spatial Dropout layer, which promotes independence between feature maps.

Empirical evaluation of the considered approach on five diverse cell and CFU segmentation datasets supports the findings and promotes investigation into multi-pathway structures, while proving the superiority of the corresponding architecture. All evaluated datasets, albeit being very different in terms of RGB palettes, pixel intensities, and textures, share some common characteristics (e.g., the presence of an agar plate) and provide a solid generalization ground for the presented model, which is capable of capturing fine-grained discriminative features across different receptive fields. The latter clearly differentiates the proposed approach from the existing state-of-the-art U-Net implementations, which deal with a single hard-coded backbone pathway. Finally, our ablation study indicates that an overall success of the proposed architecture cannot be attributed just to the presence of Spatial Dropout, which promotes generalization and feature independence.

An intriguing finding of the analysis of the multi-path U-Net model is associated with the interconnecting bottom layer, which at first performs concatenation across the channel/filter axis of all the pathways and then applies Layer Normalization. The choice of the latter is crucial for the overall success of the proposed architecture and our further research might be focused on this promising direction as well. It is anticipated that fine-tuning of the bottom-most layer’s hyperparameters or even replacing it with some other conceptually different layer, such as in [20] or [29], might considerably improve the generalization performance. Another promising future research avenue is an investigation into the number of pathways needed to achieve even better generalization. Here, it is anticipated that it is possible to make further improvements, to reach some feasible upper bound, which is linked to the input dimensions before encountering any severe overfitting. Future work will also be dedicated toward multi-head attention layers, which can play an important role in the biological and medical video analysis.

Compared to the previous studies of Isensee et al. [15], Zhou et al. [9], Ibtehaz et al. [19] and Kolařík et al. [27], the demonstrated multi-path U-Net architecture delivers better results in terms of almost all considered metrics, and it proves to be an efficient model in regard to eliminating image artifacts. While bolstering the standard U-Net architecture with multiple pathways and intertwining layers, it should be noted that it can be more difficult to understand the inner dynamics of such a system and to apply gradient-based attribution methods [37] for model explainability. The other evident limitation of the proposed approach is the necessity for all pathways to provide consistent and compatible output dimensions at the bottleneck layers, where they are coupled by means of concatenation and Layer Normalization. Finally, we must admit that without a proper multi-GPU parallelization, when nodes and operations of a TensorFlow graph are executed sequentially on one GPU, we get a near-linear increase in computational time with each additional pathway in the proposed approach compared to the “plain” U-Net architecture with a single backbone. The latter can be solved implicitly by assigning each pathway to a separate GPU card.

To wrap up the discussion section, we summarize the pros and cons of the proposed approach as follows.

1 Pros of the proposed approach:
  - Resilient multi-pathway backbone with individual receptive field pathways.
  - Better flow of visual information through a different order of resolutions in each pathway.
  - Enhanced generalization performance by means of the pathway wiring, Layer Normalization, and Spatial Dropout.
  - Possibility to introduce an implicit multi-GPU parallelization.
2 Cons of the proposed approach:
  - Necessity for all pathways to provide consistent and compatible output dimensions at the bottleneck layers.
  - Near-linear increase in computational time on a single GPU card.
  - Difficulties in the application of gradient-based attribution methods.

## 5. Conclusions

The analysis of biomedical images plays an important role in the healthcare and food safety domains. In this article, a novel deep learning architecture was developed to address several shortcomings of the original U-Net architecture. The proposed multi-path U-Net model combines individual receptive field pathways, which are merged together at the bottom-most layer by concatenation and subsequent application of Layer Normalization and Spatial Dropout. By experimental validation, better segmentation and generalization results are achieved for all evaluated datasets. In this paper, the proposed method was validated on cell and CFU segmentation tasks and proved to be a perspective approach for practical considerations in the assessment of a microbiological contamination of food samples, as well as for the monitoring of a cellular morphology, in vitro.

## Figures and Tables

**Table 1:** Dataset characteristics.

Dataset	Type	Cardinality	Image Sizes
BF-C2DL-HSC	Cell	57	1010 × 1010 × 3
BF-C2DL-MuSC	Cell	100	1036 × 1070 × 3
PhC-C2DL-U373	Cell	36	520 × 696 × 3
Fluo-N2DL-HeLa	Cell	184	700 × 1010 × 1
Proprietary CFU	CFU	150	3024 × 3024 × 3

**Table 2:** Mean segmentation results with standard deviations across all classes (background and foreground) under the Dice similarity coefficient [31,32] for U-Net++, Plain U-Net, Dense U-Net, MultiRes U-Net, and the proposed multi-path U-Net architecture with (2) and without (1) Spatial Dropout.

Architecture	Params	Dataset				
		BF-C2DL-HSC	BF-C2DL-MuSC	PhC-C2DL-U373	Fluo-N2DL-HeLa	CFU
U-Net++	9.99 M	0.8272 ± 0.0483	0.9189 ± 0.0029	0.9554 ± 0.0074	0.9763 ± 0.0001	0.8866 ± 0.0082
Plain U-Net	10.20 M	0.9501 ± 0.0054	0.9146 ± 0.0016	0.9544 ± 0.0045	0.9787 ± 0.0001	0.8919 ± 0.0059
Dense U-Net	9.68 M	0.9599 ± 0.0029	0.9186 ± 0.0020	0.9550 ± 0.0055	0.9785 ± 0.0001	0.8930 ± 0.0053
MultiRes U-Net	10.03 M	0.5317 ± 0.0261	0.6594 ± 0.0401	0.9510 ± 0.0163	0.9789 ± 0.0001	0.8664 ± 0.0180
Multi-Path U-Net ^(1)^	10.03 M	0.9498 ± 0.0062	0.9224 ± 0.0016	0.9553 ± 0.0048	0.9789 ± 0.0001	0.9007 ± 0.0044
Multi-Path U-Net ^(2)^	10.08 M	**0.9604 ± 0.0050**	**0.9238 ± 0.0013**	**0.9612 ± 0.0059**	**0.9796 ± 0.0001**	**0.9025 ± 0.0053**

**Table 3:** Segmentation results with standard deviations for the foreground class under the Dice similarity coefficient [31,32] for U-Net++, Plain U-Net, Dense U-Net, MultiRes U-Net, and the proposed multi-path U-Net architecture with (2) and without (1) Spatial Dropout.

Architecture	Params	Dataset				
		BF-C2DL-HSC	BF-C2DL-MuSC	PhC-C2DL-U373	Fluo-N2DL-HeLa	CFU
U-Net++	9.99 M	0.6554 ± 0.0965	0.8389 ± 0.0056	0.9161 ± 0.0143	0.9594 ± 0.0001	0.7784 ± 0.0158
Plain U-Net	10.20 M	0.9007 ± 0.0105	0.8302 ± 0.0030	0.9142 ± 0.0089	0.9634 ± 0.0001	0.7884 ± 0.0117
Dense U-Net	9.68 M	0.9202 ± 0.0057	0.8383 ± 0.0038	0.9151 ± 0.0108	0.9630 ± 0.0001	0.7903 ± 0.0104
MultiRes U-Net	10.03 M	0.1133 ± 0.0386	0.3630 ± 0.0712	0.9081 ± 0.0312	0.9637 ± 0.0001	0.7379 ± 0.0349
Multi-Path U-Net ^(1)^	10.03 M	0.9002 ± 0.0121	0.8457 ± 0.0030	0.9159 ± 0.0094	0.9637 ± 0.0002	0.8057 ± 0.0086
Multi-Path U-Net ^(2)^	10.08 M	**0.9214 ± 0.0098**	**0.8487 ± 0.0025**	**0.9267 ± 0.0113**	**0.9648 ± 0.0002**	**0.8090 ± 0.0103**

**Table 4:** CFU segmentation results under MAE and accuracy scores at 3 and 20 bins (for the CFU counts) for U-Net++, Plain U-Net, Dense U-Net, MultiRes, and multi-path U-Net with Spatial Dropout.

Architecture	Metrics		
	MAE	Accuracy@3	Accuracy@20
U-Net++	36.81	0.9000	0.5400
Plain U-Net	31.49	0.9000	0.5266
Dense U-Net	33.96	0.8867	0.5133
MultiRes U-Net	44.33	0.7750	0.4750
Multi-Path U-Net	**29.40**	0.8933	**0.6000**

## Data Availability

All public datasets are available at: http://celltrackingchallenge.net/2d-datasets (accessed on 1 December 2021).
